# Host species identity, site and time drive temperate tree phyllosphere bacterial community structure

**DOI:** 10.1186/s40168-016-0174-1

**Published:** 2016-06-18

**Authors:** Isabelle Laforest-Lapointe, Christian Messier, Steven W. Kembel

**Affiliations:** Département des sciences biologiques, Université du Québec à Montréal, C.P. 8888, Succ. Centre-Ville, Montréal, H3C 3P8 Québec Canada; Centre d’étude de la forêt, Université du Québec à Montréal, C.P. 8888, Succ. Centre-Ville, Montréal, H3C 3P8 Québec Canada; Institut des Sciences de la Forêt tempérée, Université du Québec en Outaouais, Ripon, J0V 1V0 Québec Canada

**Keywords:** Phyllosphere, Bacteria, Plant-bacteria interaction, Microbiome, Temperate forest

## Abstract

**Background:**

The increasing awareness of the role of phyllosphere microbial communities in plant health calls for a greater understanding of their structure and dynamics in natural ecosystems. Since most knowledge of tree phyllosphere bacterial communities has been gathered in tropical forests, our goal was to characterize the community structure and assembly dynamics of phyllosphere epiphytic bacterial communities in temperate forests in Quebec, Canada. We targeted five dominant tree species: *Acer saccharum*, *Acer rubrum*, *Betula papyrifera*, *Abies balsamea*, and *Picea glauca*. We collected 180 samples of phyllosphere communities on these species at four natural forest sites, three times during the growing season.

**Results:**

Host functional traits (i.e., wood density, leaf nitrogen content) and climate variables (summer mean temperature and precipitation) were strongly correlated with community structure. We highlight three key findings: (1) temperate tree species share a “core microbiome”; (2) significant evolutionary associations exist between groups of bacteria and host species; and (3) a greater part of the variation in phyllosphere bacterial community assembly is explained by host species identity (27 %) and species-site interaction (14 %), than by site (11 %) or time (1 %).

**Conclusions:**

We demonstrated that host species identity is a stronger driver of temperate tree phyllosphere bacterial communities than site or time. Our results suggest avenues for future studies on the influence of host functional traits on phyllosphere community functional biogeography across terrestrial biomes.

**Electronic supplementary material:**

The online version of this article (doi:10.1186/s40168-016-0174-1) contains supplementary material, which is available to authorized users.

## Background

Microorganisms colonize aerial tree surfaces (i.e., bark, leaves), enabling interactions that are essential for plant growth and fitness [1–3]. Aerial plant surfaces (mostly leaves), a habitat known as the phyllosphere, are estimated to sum up to 4 × 10^8^ km^2^ on Earth [4], which is almost equivalent to the total surface of the earth. The phyllosphere habitat is extremely poor in nutrients and exposed to a rapid and pronounced fluctuation of physical conditions [1]. Tree phyllosphere microbial communities are mainly composed of bacteria and endophytic fungi [1, 5]. These communities are extremely diverse [6–9] and contribute to host protection and productivity [10, 11]. Although our knowledge of plant-microbe interactions on tree leaf surfaces is still limited (but see [11, 12] for reviews), most studies have focused on endophytic fungi [8, 13, 14] and pathogens [15, 16] limiting our knowledge of the complex dynamics at play for other organisms. Studies of the tree phyllosphere are more and more frequent, with most studies focusing on tropical forests [17–19].

Bacteria exhibit a wide range of metabolic diversity, which allows them to survive in stressful environments where sources of energy are limited [20]. Although many aspects of phyllosphere bacterial metabolism and functional traits are poorly understood, the first censuses have revealed the presence of anoxygenic phototrophic bacteria [21]. Many bacteria abundant in the phyllosphere, such as *Methylobacterium*, have been shown to positively influence plant health and development [22, 23] mainly through the production of secondary metabolites interacting with host hormone production and influencing plant growth [11]. While high-throughput sequencing techniques provide more information on plant-bacteria interactions, there is still no clear understanding of host-bacteria association patterns across multiple host species. For example, individual trees have been shown to share part of their dominant bacterial community [18], yet little is known about this “core” microbiome, the group of bacterial taxa shared among multiple communities sampled from the same habitat [24]. Understanding the drivers of phyllosphere bacterial diversity is the first step toward developing management strategies that encourage a healthy phyllosphere microbial community structure favoring tree health and function.

Phyllosphere bacterial community composition is the result of a combination of dispersal history, host selection [9, 17], growth, and survival in the face of environmental conditions and competition [11, 25]. Hypotheses for the ecological processes structuring phyllosphere communities have included lottery models of colonization [26], as well as filtering models whereby environmental attributes act as a filter restricting the bacterial taxa that are able to persist on the leaf [27]. Although drivers of phyllosphere microbial assembly have been quantified in previous studies both for fungi [28, 29] and bacteria [9, 27, 30], most of these studies evaluated only a single potential driver of phyllosphere community structure.

In this study, we explore the ecological drivers of variation in leaf bacterial community composition of temperate trees, taking into account the influence of multiple drivers. Our objectives are (1) to identify the epiphytic bacteria present in the phyllosphere of temperate forest trees; (2) to detect the patterns of associations between host taxa and bacteria; and (3) to quantify the relative influence of three drivers on phyllosphere bacterial community composition: host species identity, site, and sampling time. We selected five common temperate tree species present at all sites to obtain a fair representation of Quebec’s temperate forests, including both angiosperms and gymnosperms: *Abies balsamea* (Balsam fir), *Acer rubrum* (Red maple), *Acer saccharum* (Sugar maple), *Betula papyrifera* (Paper birch), and *Picea glauca* (White spruce). We collected 180 samples of phyllosphere communities on these species at four natural forest sites (Additional file 1: Table S1, Figure S1), three times during the growing season. Bacterial community structure was determined through High-throughput Illumina sequencing of the bacterial 16S rRNA gene [31].

## Results

### Sequences, OTUs, and taxonomy

Sequencing identified 15,873 bacterial operational taxonomic units (OTUs, sequences binned at 97 % similarity) in phyllosphere samples, an average of 517 ± 16 OTUs (mean ± standard error) per tree sampled. Most of these bacterial taxa were rare, with 52.6 % of bacterial OTUs occurring only on a single tree. Each tree sampled revealed additional bacterial taxa as shown by a collector’s curve of the number of OTUs per sample (Additional file 1: Figure S2). Four of the nine most abundant bacterial classes belonged to the phylum *Proteobacteria*: *Alpha*- (68 % of all sequences), *Beta-* (6 %), *Gamma-* (5 %), and *Deltaproteobacteria* (3 %); three belonged to the phylum *Bacteroidetes*: *Cytophagia* (4 %), *Sphingobacteria* (1 %), and *Saprospirae* (1 %); and finally the classes *Acidobacteria* (6 %) and *Actinobacteria* (5 %) were also abundant.

We detected a “core microbiome” [24], defined as OTUs present on 99 % or more of all trees sampled, of 19 bacterial OTUs belonging to two phyla, four classes, and seven families. This core microbiome represented less than 0.001 % of the bacterial taxonomic diversity but more than 42.7 % of sequences (Additional file 1: Table S2). The most abundant core microbiome OTUs included representatives of *Methylocystaceae* (two OTUs at 17.8 and 4 % relative abundance), *Beijerinckia* (two OTUs at 4.0 and 1.2 %), *Sphingomonas* (two OTUs at 2.4 and 1.2 %), *Acidobacteriaceae* (2.3 %), *Oxalobacteraceae* (2.3 %), and *Acetobacteraceae* (1.2 %) (Additional file 1: Table S2). Most of the abundant OTUs showed significant associations with host species identity, site, and sampling time (Table 1).

**Table 1:** Linear models of the relationship between each of core microbiome OTU abundance and the drivers

Taxonomy (family)	OTU number	Time		Site			Host species				Model total R^2^ (%)
		July	August	Bic	Gatineau	Sutton	ACRU	ACSA	BEPA	PIGL	
Acetobacteraceae	3293	NS	NS	NS	NS	NS	NS	NS	−0.71**	−0.53*	18
	7913	NS	NS	−0.80**	−1.77***	−1.12***	NS	−0.71**	0.66**	−0.91***	45
	20300	NS	NS	NS	NS	NS	−1.04***	−0.49*	−2.19***	NS	46
	30571	NS	NS	NS	−1.91***	NS	NS	NS	−0.79**	−1.01***	58
	33295	NS	NS	NS	NS	NS	0.6777*	NS	−0.68*	NS	19
Acidobacteriaceae	4366	NS	NS	−1.01***	−1.42***	−1.06***	NS	−1.17***	−0.99***	−0.84**	32
	30762	NS	NS	−0.94***	−1.06***	−0.63***	NS	−1.09***	−0.91***	−0.70**	30
	37541	NS	NS	−1.47***	−2.47***	−0.77**	1.33***	NS	1.30***	−0.99***	55
	42054	NS	0.51*	−0.71**	−1.31***	−0.56*	−1.55***	−2.02***	−0.68*	−0.72**	44
	45264	NS	NS	NS	−1.72***	−0.58**	−1.61***	−1.80***	−1.78***	−0.52*	60
Beijerinckiaceae	17267	NS	NS	−0.55*	−0.97***	−0.66**	1.60***	0.74**	NS	NS	39
	43328	NS	NS	NS	−0.74**	NS	0.92***	NS	NS	NS	26
Cystobacterineae	45353	−0.67**	NS	−1.68***	−1.69***	−1.54***	1.61***	NS	1.72***	NS	50
Methylocystaceae	6292	NS	NS	NS	−0.66**	NS	1.24***	NS	NS	−0.49*	34
	32918	NS	NS	0.68*	−1,45***	NS	−1,83***	−1,70***	−2,29***	−0,69*	55
	38758	NS	NS	NS	−0.72**	NS	1.28***	0.67**	NS	NS	38
Oxalobacteraceae	26524	NS	NS	NS	NS	NS	1.53***	1.95***	NS	NS	32
Sphingomonadaceae	11233	NS	0.81**	NS	0.99**	0.99**	NS	NS	−1.96***	NS	42
	20227	NS	NS	−0,88**	−1,26***	−1,36***	NS	NS	NS	NS	22

Numbers represent the coefficient of factors. Significance levels for each variable are given by: *p < 0.05; **p < 0.01; ***p < 0.001; NS, *p* > 0.1

### Biomarker analysis

**Fig. 1:**
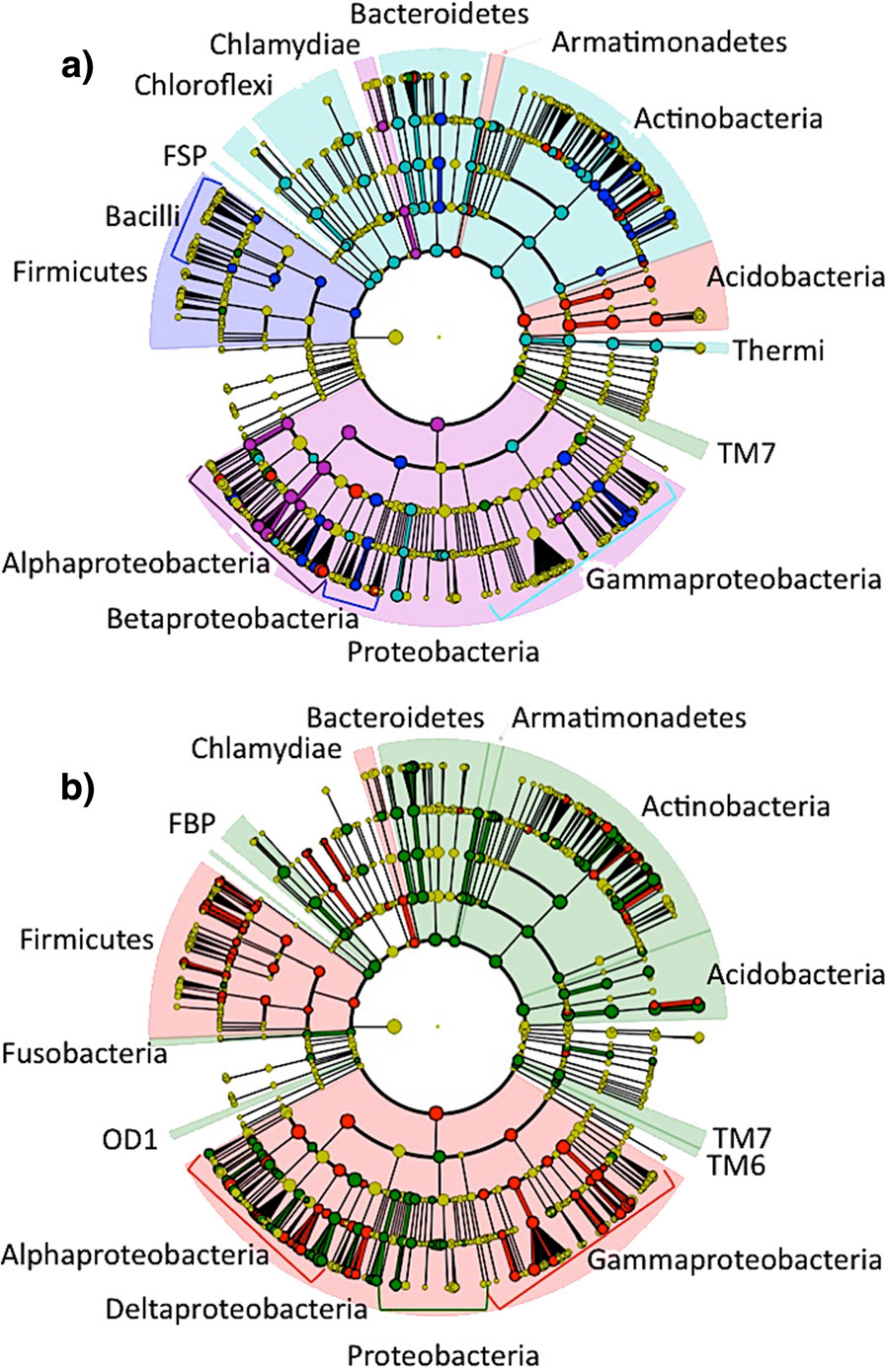
Cladogram of significant associations between phyllosphere bacterial taxon and host identity (linear discrimination algorithm LEfSe). Legend: **a** *color* indicates association with a host species (*green*: *Acer rubrum*; *blue*: *Acer saccharum*; *purple*: *Betula papyrifera*; *red*: *Abies balsamea*; *turquoise*: *Picea glauca*), **b** *green* indicates an association with gymnosperms (*Abies balsamea* and *Picea glauca)*, and *red* with the angiosperms (*Acer rubrum*, *Acer saccharum,* and *Betula papyrifera*). The *circles*, *parentheses*, and *shading* indicate with which host-group the bacterial taxonomic group is associated

At the OTU level, four OTUs were significantly associated with host species: two OTUs from *Acetobacteraceae* associated with both conifer species; one OTU from *Cystobacterineae* associated with *Acer saccharum*; and finally one OTU from *Rickettsiaceae* associated with *Acer rubrum* (Table 1, Additional file 1: Table S3). At the species level, 147 bacterial species were significantly associated with host species (Fig. 1a, Additional file 1: Table S3). Overall, the *TM7* group was significantly associated with *Acer rubrum*; the *Firmicutes*, *Bacilli*, and *Betaproteobacteria* were associated with *Acer saccharum*; the *Proteobacteria*, *Alphaproteobacteria* and *Chlamydiae* with *Betula papyrifera*; the *Armatimonadetes* and *Acidobacteria* with *Abies balsamea*; and finally, the *Actinobacteria*, *Bacteroidetes*, *Chloroflexi* and *FSP* were significantly associated with *Picea glauca*. At a broader taxonomic scale, 129 bacterial species were significantly associated with the gymnosperms and 79 with the angiosperms (Fig. 1b, Additional file 1: Table S4). In short, the *Armatimonadetes*, *Actinobacteria*, *Bacteroidetes*, *Acidobacteria*, *TM7*, *TM6*, *Deltaproteobacteria*, *OD1*, *Fusobacteria*, and *FBP* were associated with the gymnosperms; whereas the groups *Chlamydiae*, *Proteobacteria*, *Gammaproteobacteria*, *Alphaproteobacteria*, and *Firmicutes* were associated with angiosperms.

### Drivers of variation in phyllosphere bacterial community composition and diversity

**Fig. 2:**
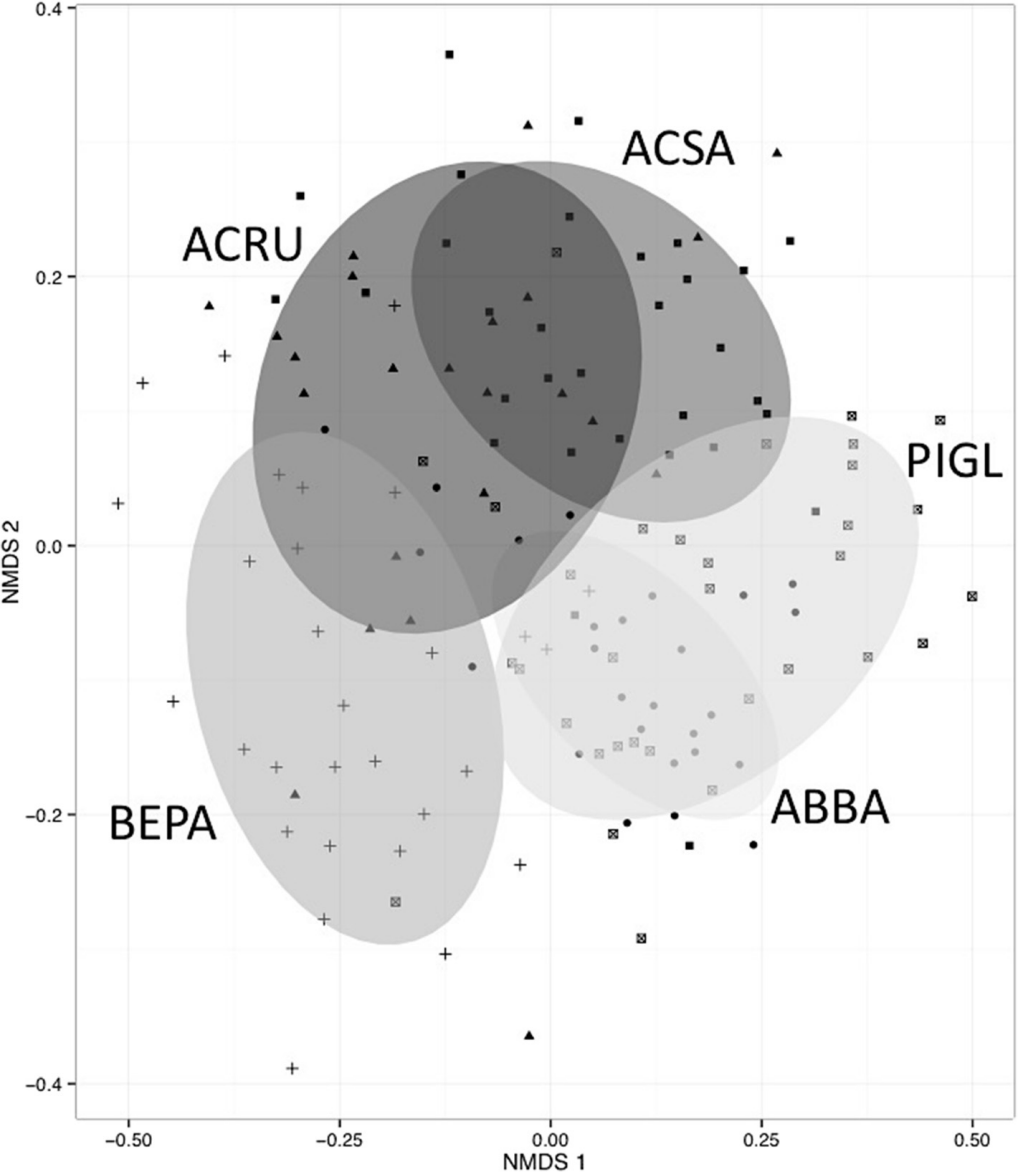
Non-metric multidimensional scaling (NMDS) ordination of variation in bacterial community structure of temperate tree phyllosphere. Legend: Ordination based on Bray-Curtis distances among samples. Samples (*points*) are shaded based on host species identity (*ABBA* for *Abies balsamea*; *ACRU* for *Acer rubrum*; *ACSA* for *Acer saccharum*; *BEPA* for *Betula papyrifera*; and *PIGL* for *Picea glauca*); *ellipses* indicate 1 standard deviation confidence intervals around samples from each host species

**Fig. 3:**
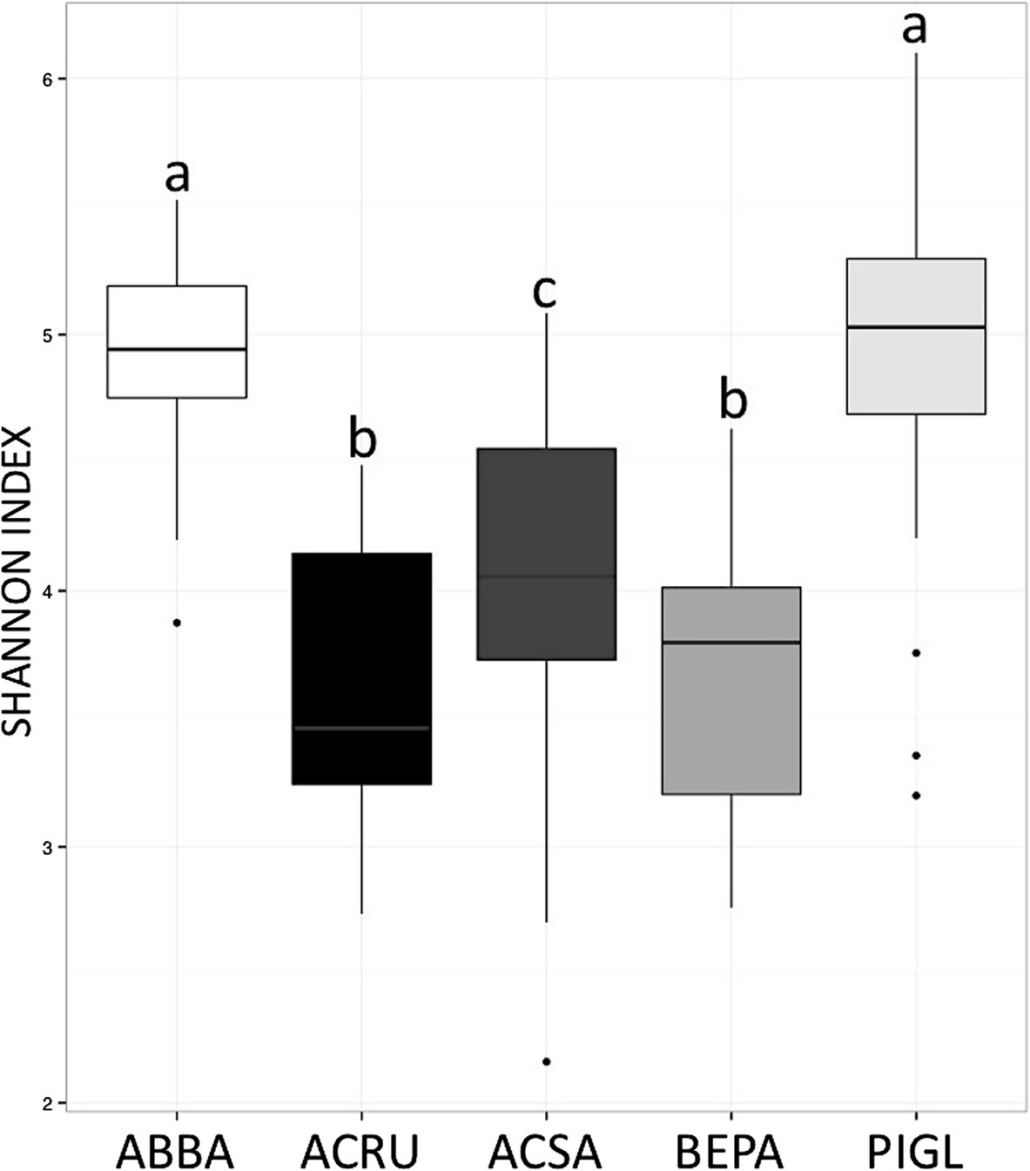
Shannon diversity indices of phyllosphere bacterial communities for different host species. Legend: *Boxplots* are shaded by host species (*ABBA* for *Abies balsamea*; *ACRU* for *Acer rubrum*; *ACSA* for *Acer saccharum*; *BEPA* for *Betula papyrifera*; and *PIGL* for *Picea glauca*). *Letters* indicate the results of a post-hoc test of Tukey multiple comparisons of means at a 95 % family-wise confidence level between host species. Only the pairs BEPA-ACRU and PIGL-ABBA are not significantly different

An analysis of variation in community structure (PERMANOVA on Bray-Curtis distances) explained by different factors showed that gymnosperm/angiosperm groups explained 13.4 % (*p* = 0.001), host taxonomic family explained 9.3 % (*p* = 0.001); host genus explained 2.21 % (*p* = 0.002), and finally host species explained 2.1 % (*p* = 0001). Host taxonomic levels thus explained 24.8 % of the variation in phyllosphere bacterial community structure. Host species identity, the interaction between species and site, site, and time, explained, respectively, 27.2, 13.8, 10.9, and 1.5 % of the variation in leaf bacterial community structure (PERMANOVA on Bray-Curtis distances) for a total of 53.4 % (Fig. 2 and Table 2). These factors showed similar trends when explaining the variation in leaf bacterial phylogenetic community structure (PERMANOVA on weighted UniFrac distances) thus here we present only the results of analyses based on Bray-Curtis dissimilarities. The best model from the linear mixed models of variation in bacterial alpha diversity explained by different factors (model: Shannon Diversity ~ (1|TREE) + Species + Site + Time; fit by maximum likelihood) showed that tree identity explains 13 % of the variance in bacterial community alpha diversity (∆AIC = 1.2). Only species, site, and their interactions significantly affected microbial diversity. The Abitibi site was significantly less diverse than the three other sites. Conifer species (*Pinus* and *Abies*) showed a significantly higher alpha diversity than the three deciduous species (Fig. 3).

**Table 2:** Bacterial community structure variation explained by various factors (PERMANOVA on Bray-Curtis dissimilarities)

Variables		Bray-Curtis dissimilarities	
		*R* ^2^ (%)	Pr (>*F*)
Single factor	Host species	27.2	0.001^***^
	Site	10.9	0.001^***^
	Time	1.5	0.008^**^
Second order interaction	Host species*site	13.8	0.001^***^
	Site*time	NS	NS
Third order interaction	Host species*site*time	NS	NS

The model explained 53.4 %. Significance levels for each variable are given by: *p < 0.05; **p < 0.01; ***p < 0.001; NS, *p* > 0.1

**Fig. 4:**
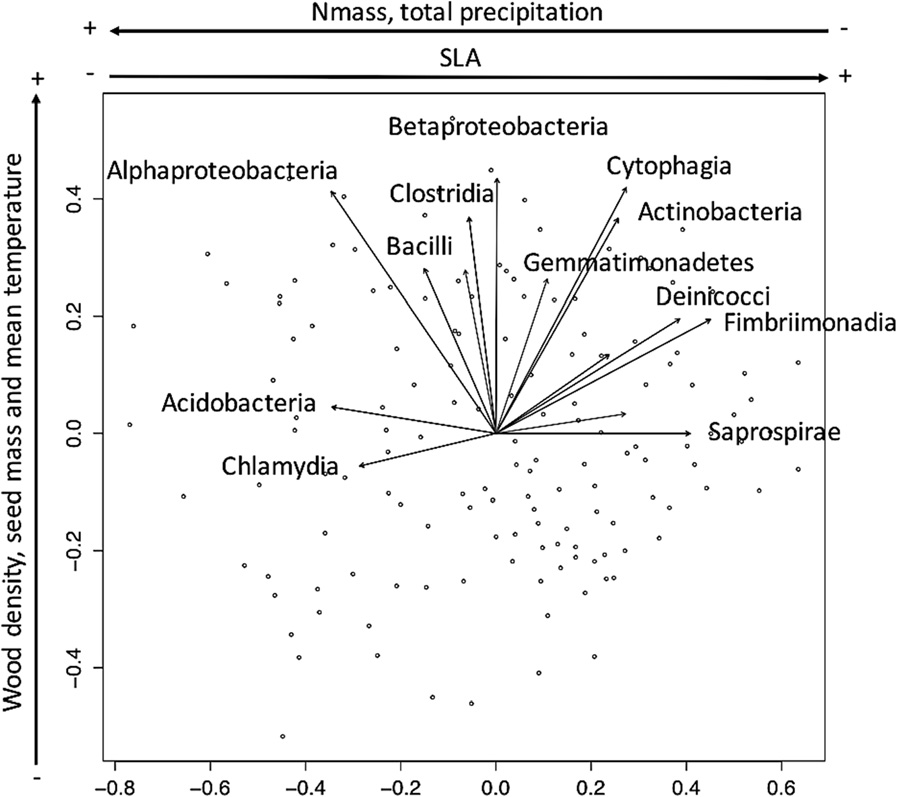
Non-metric multidimensional scaling (NMDS) ordination of variation in bacterial community structure of temperate tree phyllosphere. Legend: Ordination based on Bray-Curtis dissimilarities among samples. *Points* represent samples and *arrows* inside plot margins represent the significant (*p* < 0.001) correlations between NMDS axes vs. the relative abundances of bacterial classes in communities. *Arrows* outside plot margins indicate host plant traits and climatic variables with significant (*p* < 0.007 for functional traits and *p* < 0.025 for climatic data) correlations with sample scores on each ordination axis

Four functional traits were significant drivers of phyllosphere bacterial community structure (PERMANOVA on Bray-Curtis distances): nitrogen content of leaves (N_mass_; *p* = 0.001), specific leaf area (SLA; *p* = 0.001), wood density (WD, *p* = 0.001) and seed mass (S_mass_; *P* = 0.001). The relative abundances of *Acidobacteria*, *Chlamydia*, *Deinococci*, *Fimbriimonadia*, and *Saprospirae* were significantly correlated (*p* < 0.001) with traits related to the leaf economics spectrum (N_mass_ and SLA). These bacterial classes were more abundant on the leaves of tree species that have lower leaf nitrogen concentrations and higher leaf dry matter content (Fig. 4). The relative abundances of *Actinobacteria*, *Alphaproteobacteria*, *Bacilli*, *Betaproteobacteria*, *Clostridia*, *Cytophagia*, and *Gemmatimonadetes* were significantly correlated (*p* < 0.001) with traits related to wood density (Fig. 4). Climate variables were weakly but significantly correlated with phyllosphere bacterial community structure (total precipitation: 1.8 % of variance explained (*p* < 0.002), mean monthly temperature: 1.2 % of variance explained (*p* < 0.006)).

## Discussion

In terms of the taxonomic composition of phyllosphere communities, temperate leaf communities seem to differ slightly from past reports of tropical and temperate phyllosphere community structure. Natural temperate phyllosphere communities in Quebec forests were dominated by *Alphaproteobacteria* (68 % of all sequences), contrasting with 27 % [17] and 22.8 % [18] of sequences in tropical tree species and 24.5 % in suburban temperate stands [9]. Due to the necessity of using chloroplast-excluding primers to prevent contamination of samples by plant DNA [32], we were unable to quantify the abundance of Cyanobacteria in the temperate forest phyllosphere. However, metagenomic studies have demonstrated that Cyanobacteria are typically rare in the vascular plant phyllosphere [11, 33], and by using the same chloroplast-excluding 16S primer employed by previous studies [9, 18, 25], we were able to eliminate primer taxonomic bias as an explanation of differences in clade abundances among studies.

In contrast with Redford et al., [9], we detected the presence of a core phyllosphere microbiome, a group of bacterial taxa shared among multiple communities sampled from the same habitat and thought to play key ecological roles [24]. The core microbiome was composed of 19 OTUs representing 42.7 % of all sequences present in more than 99 % of samples, even when study sites were hundreds of kilometers apart. Assuming that bacterial OTUs represent ecologically or evolutionarily coherent units [34], this finding suggests that bacteria from a similar metacommunity colonize tree leaves across Quebec’s temperate forests by dispersal through a variety of vectors (i.e., air, rain, soil) [35], homogenizing the epiphytic phyllosphere community structure across broad geographic distances.

Despite the presence of a core microbiome of abundant taxa, individual trees also showed unique communities that varied predictably across species, sites and time, suggesting a role for selection- or niche-based mechanisms during community assembly. Linear models testing the association between core microbiome OTUs vs. host species identity, site, and time explained 18 to 60 % of the variation in phyllosphere bacterial community structure (Table 1), confirming these three drivers’ roles in shaping phyllosphere community structure. In addition, biomarker analyses confirmed the existence of host selective mechanisms on phyllosphere community structure as shown by associations between numerous bacterial taxa and different host species and sites (Fig. 1).

At the tree species level, *Abies balsamea* (balsam fir) tended to associate with the order *Sphingomonadales*, as with the families *Acidobacteraceae, Solibacteraceae*, and *Frankiaceae.* The three first groups mentioned above are common in soils [36, 37], and the *Frankiaceae* are nitrogen-fixing bacteria that colonize plant roots [38]. This finding is in line with other studies showing that conifers select a different microbiome than other plant species: for example they harbor less ice nuclei active bacteria [39]. In contrast, *Betula papyrifera* (paper birch) was associated with the family *Rhodospirillaceae* (*Rhodospirillales: Alphaproteobacteria*). This bacterial family is mostly composed of purple nonsulfur bacteria that produce energy through photosynthesis [40]. Photosynthesis could be a key adaptation to the phyllosphere habitat, an environment where simple carbon sources are scarce and highly variable [1, 11]. Tree-bacteria associations were also observed at the angiosperm vs. gymnosperm level (Fig. 3), likely driven by the influence of the numerous plant functional trait differences between these clades (Lambais et al. [41] and Kembel et al. [18]).

Host species identity was the main driver of phyllosphere bacterial community structure among trees (*R*^2^ = 27 %) when compared to site and time. As shown in other studies, each tree species harbors a distinctive phyllosphere bacterial community [9, 17, 41], but our results highlight for the first time the relative influence of site (*R*^2^ = 11 % for site alone and *R*^2^ = 14 % for site species interaction) and time (*R*^2^ = 1 %) for multiple tree species. In accordance with the findings of Kembel et al. [18] in tropical forests, temperate phyllosphere epiphytic bacterial community structure was correlated with both traits linked to plant-resource uptake strategies such as leaf nitrogen content and leaf mass per area [42], and traits linked to the wood density/growth/mortality tradeoff such as wood density [43]. This confirms that phyllosphere bacterial communities are shaped by the ecological strategies of their plant hosts. These similarities also suggest that the factors driving the functional biogeography of plant-microbe associations in the phyllosphere are similar across temperate and tropical biomes, as we found a similar set of traits influencing phyllosphere community structure in temperate forests vs. those described for tropical forests [18]. Although many insights have been gained from individual tree microbiome studies in tropical and temperate biomes, meta-analyses controlling for methodological differences will be needed to better understand plant-microbe associations across terrestrial biomes and environmental gradients.

Consistent with the idea of environmental selective pressure on phyllosphere communities due to abiotic conditions such as temperature and precipitation, climate differences between sites (monthly precipitation and mean monthly temperature) were correlated with variation in phyllosphere bacterial community structure. In addition, the effect of sampling time and the interaction between sampling time and site on phyllosphere community structure suggests that phyllosphere communities undergo a succession during the growing season. As previously demonstrated for individual host tree species by Redford and Fierer [25] for bacterial communities and by Jumpponen and Jones [7] for fungal communities, leaf communities were temporally dynamic. However, the variance explained by sampling time was small relative to the importance of host species and site, suggesting that once a community of bacteria successfully colonizes a leaf, temporal changes are not enough to overcome the influence of host species identity and site on community assembly. In the temperate forest we studied, growing season had a significant impact on community structure at two sites at the beginning and end of the growing season: the months of June and August. To minimize phyllosphere community structure variation due to sampling time, leaf sampling in these forests should be completed in July once leaves are fully mature but before senescence begins in August.

We found consistent evidence that community composition and alpha diversity differed between coniferous (gymnosperm) vs. broadleaved (angiosperm) tree species. Our results show that several functional traits characteristic of tree ecological strategy explained differences in leaf community structure. However, additional leaf functional traits not measured here (i.e., increased leaf cuticle thickness and wax composition of gymnosperms) could also play a key role by limiting carbon compound availability and humidity at the leaf surface [9, 11]. Because our sampling did not exclusively target the new needles of conifers, a study of succession on conifer needles will be needed to determine if the diversity is due to the particular selective power of the host species or to the longer accumulation through leaf life span of the bacterial community on conifer leaves.

## Conclusions

In this study, we describe for the first time natural temperate tree phyllosphere bacterial communities across multiple tree species while exploring the influence of host species identity, site, and time of sampling on phyllosphere community structure. In addition, we performed the first simultaneous evaluation of the importance of key dispersal-related and niche-based drivers such as host species identity (phylogeny, co-evolution, functional traits), geographical location (dispersal history and abiotic conditions) and time of sampling (abiotic conditions) on tree phyllosphere bacterial communities. Our key findings include: (1) that temperate host species share a “core microbiome”; (2) that there are significant associations between groups of bacteria and host species; and finally (3) that a greater part of the variation in phyllosphere bacterial community assembly is explained by host species identity rather than by site or time.

## Methods

### Study site

The study plots are located in four natural temperate forest stands in Quebec (Additional file 1: Table S1): Sutton (45° 6' 46" N; 72° 32' 28" W), Abitibi (48° 9' 45" N; 79° 24' 4" W), Gatineau (45° 44' 50" N; 75° 17' 57" W), and Bic (48° 20' 1" N; 68° 49' 3" W). Distances between sites range from 295 km (Sutton and Gatineau) to 765 km (Abitibi and Bic) (Additional file 1: Figure S1). This region is characterized by a cold and humid continental climate with temperate summer. We obtained monthly climate data from Canada’s public weather database [44] (Additional file 1: Table S1).

### Bacterial community collection

We sampled at each site three times during the 2013 growing season (June, July, and August) from three individuals for each tree species, a total of 180 samples. For each randomly chosen tree, we clipped 50–100 g of shade leaves at mid-canopy height (1–2 m above the bottom of the tree’s canopy) into sterile roll bags with surface-sterilized shears. For bacterial community collection and amplification, we used the protocols described by Kembel et al. [18]. We collected microbial communities from the leaf surface by agitating the samples in a diluted Redford buffer solution. We resuspended cells in 500 μL of PowerSoil bead solution (MoBio, Carlsbad, California). We extracted DNA from isolated cells using the PowerSoil kit according to the manufacturer’s instructions and stored at −80 °C.

### DNA library preparation and sequencing

We used a two-stage PCR approach to prepare amplicon libraries for the high-throughput Illumina sequencing platform. The use of combinatorial primers for paired-end Illumina sequencing of amplicons reduced the number of primers while maintaining the diversity of unique identifiers [45]. First, to avoid PCR contamination by chloroplast DNA amplification, we targeted the V5–V6 region of the bacterial 16S rRNA gene using cyanobacteria-excluding primers (16S primers 799 F-1115R [9, 25, 46]) following protocols described by Kembel et al. [18]. These chloroplast-excluding primers have been widely used in studies of phyllosphere bacteria in order to avoid contamination by host plant DNA [32], and their use is justified since while they exclude both plant chloroplasts and cyanobacteria sequences, cyanobacteria are known to be rare in tree phyllosphere communities [11, 33]. Using cleaned PCR product as a template, a second PCR was performed with custom HPLC-cleaned primers to further amplify 16S products and complete the Illumina sequencing construct (PCRII_for: 5′-AAGCAGAAGACGGCATACGAGATCGGTCTCGGCATTCCTGC; PCRII_rev: 5′-ATGATACGGCGACCACCGAGATCTACACTCTTTCCCTACACGACG). We cleaned the resulting product using MoBio UltraClean PCR cleanup kit. We isolated a ~445-bp fragment by electrophoresis in a 2 % agarose gel, and recovered DNA with the MoBio GelSpin kit. We prepared multiplexed 16S libraries by mixing equimolar concentrations of DNA, and sequenced the DNA library using Illumina MiSeq 250-bp paired-end sequencing at Genome Quebec.

We processed the raw sequence data with PEAR [47] and QIIME [48] pipelines to merge paired-end sequences to a single sequence of length of approximately 350 bp, eliminate low quality sequences (mean quality score < 30 or with any series of 5 bases with a quality score < 30), and de-multiplex sequences into samples. We eliminated chimeric sequences using the Uclust and Usearch algorithms [49]. Then, we binned the remaining sequences into operational taxonomic units (OTUs) at a 97 % sequence similarity cutoff. We determined the taxonomic identity of each OTU using the BLAST algorithm and Greengenes database [50] as implemented in QIIME [48].

### Host plant trait data

We obtained data on host plant functional traits (Additional file 1: Table S5) including drought tolerance (D_tol_), average maximum height (H_max_), leaf nitrogen mass (N_mass_), seed mass (S_mass_), shade tolerance (S_tol_), specific leaf area (SLA), and wood density (WD) from a global database collected by Abrams and Kubiske [51], Burns and Honkala [52], Farrar [53], Shipley and Vu [54], Wright et al. [42], Niinemets and Valladares [55], Chave et al. [56], and USDA [57].

### Biomarker analysis

We tested for the significant associations between bacterial taxa and host species, host taxonomy (angiosperms vs. gymnosperms), and sites using the linear discriminant analysis effect size (LEfSe) algorithm [58]. The LEfSe algorithm aims to discover biomarkers (genes, pathways, or taxa) of different sample groups employing the linear discriminant analysis to approximate the effect size of each biomarker identified. A significant association between bacterial clades and a specific group (i.e., a host tree species) will be detected when there is consistently higher relative abundance of the clade in the group’s samples. Among the bacterial clades detected as statistically and biologically relevant, the strongest scores identify which clades have the greatest explanatory power for differences between communities [58].

### Statistical analyses

Because PCR and sequencing errors could lead to spurious OTU identification [59], we created a database excluding OTUs represented by less than 20 sequences to eliminate rare OTUs. Analyses were performed on both the full database and the database with rare OTUs excluded to assess the results’ sensibility to rarefaction. The number of sequences per sample ranged from 4574 to 86,280. From a database of 3,868,892 quality sequences, we rarefied each sample to 4000 sequences, with 38 samples excluded from subsequent analyses due to insufficient sequence reads as a result of extraction or sequencing errors, totalizing 668,000 sequences from 142 samples representing five tree species. Rarefaction and all subsequent statistical analyses were repeated 100 times. Results did not differ qualitatively across iterations of the rarefaction and we therefore present only the result of a single random rarefaction. We performed analyses with the ape [60], ggplot2 [61], picante [62], and vegan [63] packages in R [64].

We quantified the phylogenetic variation in bacterial community structure among samples with the weighted UniFrac index, an abundance-weighted measure of the phylogenetic differentiation among bacterial communities [65]. To illustrate patterns of bacterial community structure, we performed a non-metric multidimensional scaling (NMDS) ordination of Bray-Curtis dissimilarities and weighted UniFrac distances among all samples. We identified relationships between bacterial community structure, host species identity, time, and site by conducting a permutational multivariate analysis of variance (PERMANOVA, [66]) on the community matrix. We identified functional traits and climate variables that are significant drivers of leaf community structure through a PERMANOVA. We employed a blocking randomization to account for the non-independence of observations across species and sites. The functional trait PERMANOVA was blocked by site and the climate variable PERMANOVA was blocked by species to correct for the absence of intra-site and intra-specific variation in our trait and climate data. To visualize the changes in bacterial communities with respect to different variables, we tested for correlations between these variables and community scores on the NMDS ordination axes while applying the Bonferroni correction for multiple comparisons to our significance threshold [67, 68]. The cutoffs for significant correlations (*α* = 0.05) were adjusted to *p* < 0.007 (functional traits) and *p* < 0.025 (climate data). To quantify the influence of host taxonomic levels on bacterial community structure, we performed a nested PERMANOVA (levels: angiosperm/gymnosperm, family, genus, species).

We estimated phyllosphere bacterial alpha diversity using the Shannon index calculated from OTU relative abundances for each community. We performed an analysis of variance (ANOVA) and subsequent post-hoc Tukey’s tests to test for differences in diversity across species, time, and site. To account for the repeated measures taken on individual trees in our data, we constructed a linear mixed model fitted by maximum likelihood. This model aimed to estimate the power of tree identity as a random factor in driving microbial community diversity in comparison with host species identity, site and, sampling time.

## Abbreviations

*ANOVA* analysis of variance, *D*_*tol*_ drought tolerance, *H*_*max*_ average maximum height, *LEfSe* linear discriminant analysis effect size, *N*_*mass*_, leaf nitrogen mass, *NMDS* non-metric multidimensional scaling, *OTU* operational taxonomic unit, *PERMANOVA* permutational analysis of variance, *SLA* specific leaf area, *S*_*mass*_ seed mass, *S*_*tol*_ shade tolerance, *WD* wood density

## Additional file

Additional file 1: Table S1. Description of the four study sites during the summer of 2013 (Canadian historical climate data, http://climate.weather.gc.ca/). **Table S2.** Taxonomic identity of the 19 core microbiome OTUs across the 142 trees sampled. Taxonomic identification was based on a BLAST against the greengenes database with a minimum cutoff of 50 % confidence required for assignment to a given taxonomic group. **Table S3.** Significative associations between bacterial taxonomic groups (a-Phylum, b-Class, c-Order, d-Family and e-OTUs) and tree species (LEfSe analyses). Scores identify which clades have the greatest explanatory power on differences between communities. **Table S4.** Significative associations between bacterial taxonomic groups (a-Phylum, b-Class, c-Order, d-Family and e-OTUs) with tree species classified between angiosperms and gymnosperms (LEfSe analyses). Scores identify which clades have the greatest explanatory power on differences between communities. **Table S5.** Taxonomic and functional trait information of the five tree species used in this study. Sources for functional trait information are described in the main text. **Figure S1.** Location of the four sites sampled during summer 2013 across the temperate forest of Quebec’s province. **Figure S2.** Collector’s curve (mean 95 % confidence interval) of bacterial phyllosphere operational taxonomic units (OTUs; 97 % sequence similarity cut-off) richness versus number of trees sampled in the temperate forest in 2013. (DOC 1640 kb)
