# Supplementary material for: Host species identity, site and time drive temperate tree phyllosphere bacterial community structure
Source: Microbiome. 2016 Jun 18;4:27. doi: 10.1186/s40168-016-0174-1 (PMC4912770; doi:10.1186/s40168-016-0174-1)
Supplement: Additional file 1: Table S1. — Description of the four study sites during the summer of 2013 (Canadian historical climate data, http://climate.weather.gc.ca/). Table S2. Taxonomic identity of the 19 core microbiome OTUs across the 142 trees sampled. Taxonomic identification was based on a BLAST against the greengenes database with a minimum cutoff of 50 % confidence required for assignment to a given taxonomic group. Table S3. Significative associations between bacterial taxonomic groups (a-Phylum, b-Class, c-Order, d-Family and e-OTUs) and tree species (LEfSe analyses). Scores identify which clades have the greatest explanatory power on differences between communities. Table S4. Significative associations between bacterial taxonomic groups (a-Phylum, b-Class, c-Order, d-Family and e-OTUs) with tree species classified between angiosperms and gymnosperms (LEfSe analyses). Scores identify which clades have the greatest explanatory power on differences between communities. Table S5. Taxonomic and functional trait information of the five tree species used in this study. Sources for functional trait information are described in the main text. Figure S1. Location of the four sites sampled during summer 2013 across the temperate forest of Quebec’s province. Figure S2. Collector’s curve (mean 95 % confidence interval) of bacterial phyllosphere operational taxonomic units (OTUs; 97 % sequence similarity cut-off) richness versus number of trees sampled in the temperate forest in 2013. (DOC 1640 kb) [file 40168_2016_174_MOESM1_ESM.doc]

SUPPLEMENTARY INFORMATION

TITLE: Host Species Identity, Site and Time Drive Temperate Tree Phyllosphere Bacterial Community Structure

Isabelle Laforest-Lapointe^1,2^*, Christian Messier^1,2,3^ and Steven W. Kembel^1,2^

1 Département des sciences biologiques, Université du Québec à Montréal, Montréal, (H3C 3P8), Québec, Canada

^2^ Centre d’étude de la forêt, Université du Québec à Montréal, Montréal, (H2X 3Y7), Québec, Canada

^3^ Institut des Sciences de la Forêt tempérée, Université du Québec en Outaouais, Ripon, (J0V 1V0), Québec, Canada

* Correspondence: Isabelle Laforest-L., Centre for Forest Research, Université du Québec à Montréal, Département de Sciences Biologiques, 141 av. Président-Kennedy, Montréal, (H2X 3Y7), Québec, Canada. Phone: 514-987-3000 (6936). Email: isabelle.laforest.lapointe@gmail.com

**Table S1.** Description of the four study sites during the summer of 2013 (Canadian historical climate data, <http://climate.weather.gc.ca/>).

| **Site** | **Elevation (m)** | **Month** | **Mean monthly temperature (°C)** | **Monthly precipitation (mm)** |
| --- | --- | --- | --- | --- |
| Sutton | 650 | June | 16.0 | 146.2 |
|  |  | July | 20.0 | 100.4 |
|  |  | August | 17.5 | 124.6 |
| Abitibi | 321 | June | 14.8 | 71.2 |
|  |  | July | 18.5 | 52.8 |
|  |  | August | 16.7 | 41.8 |
| Gatineau | 100 | June | 17.8 | 107.4 |
|  |  | July | 21.3 | 54.7 |
|  |  | August | 19.1 | 68.6 |
| Bic | 254 | June | 14.1 | 180.8 |
|  |  | July | 20.1 | 25.0 |
|  |  | August | 17.7 | 41.8 |

| **OTU ID** | **DOMAIN** | **PHYLUM** | **CLASS** | **ORDER** | **FAMILY** | **SEQUENCES** | **PROPORTION (%)** |
| --- | --- | --- | --- | --- | --- | --- | --- |
| denovo38758 | Bacteria | Proteobacteria | Alphaproteobacteria | Rhizobiales | Methylocystaceae | 691235 | 17,9 |
| denovo43328 | Bacteria | Proteobacteria | Alphaproteobacteria | Rhizobiales | Beijerinckiaceae | 201341 | 5,2 |
| denovo6292 | Bacteria | Proteobacteria | Alphaproteobacteria | Rhizobiales | Methylocystaceae | 154426 | 4,0 |
| denovo11233 | Bacteria | Proteobacteria | Alphaproteobacteria | Sphingomonadales | Sphingomonadaceae | 93018 | 2,4 |
| denovo37541 | Bacteria | Acidobacteria | Acidobacteriia | Acidobacteriales | Acidobacteriaceae | 89142 | 2,3 |
| denovo26524 | Bacteria | Proteobacteria | Betaproteobacteria | Burkholderiales | Oxalobacteraceae | 88783 | 2,3 |
| denovo20227 | Bacteria | Proteobacteria | Alphaproteobacteria | Sphingomonadales | Sphingomonadaceae | 47281 | 1,2 |
| denovo30571 | Bacteria | Proteobacteria | Alphaproteobacteria | Rhodospirillales | Acetobacteraceae | 45477 | 1,2 |
| denovo20300 | Bacteria | Proteobacteria | Alphaproteobacteria | Rhodospirillales | Acetobacteraceae | 34051 | 0,9 |
| denovo7913 | Bacteria | Proteobacteria | Alphaproteobacteria | Rhodospirillales | Acetobacteraceae | 32548 | 0,8 |
| denovo42054 | Bacteria | Acidobacteria | Acidobacteriia | Acidobacteriales | Acidobacteriaceae | 20341 | 0,5 |
| denovo33295 | Bacteria | Proteobacteria | Alphaproteobacteria | Rhizobiales | Methylocystaceae | 20294 | 0,5 |
| denovo45353 | Bacteria | Proteobacteria | Deltaproteobacteria | Myxococcales | Cystobacterineae | 20001 | 0,5 |
| denovo34795 | Bacteria | Proteobacteria | Alphaproteobacteria | Rhodospirillales | Acetobacteraceae | 19796 | 0,5 |
| denovo3293 | Bacteria | Proteobacteria | Alphaproteobacteria | Rhodospirillales | Acetobacteraceae | 17600 | 0,5 |
| denovo4366 | Bacteria | Acidobacteria | Acidobacteriia | Acidobacteriales | Acidobacteriaceae | 16530 | 0,4 |
| denovo17267 | Bacteria | Proteobacteria | Alphaproteobacteria | Rhizobiales | Beijerinckiaceae | 15780 | 0,4 |
| denovo45264 | Bacteria | Acidobacteria | Acidobacteriia | Acidobacteriales | Acidobacteriaceae | 12961 | 0,3 |
| denovo30762 | Bacteria | Acidobacteria | Acidobacteriia | Acidobacteriales | Acidobacteriaceae | 10934 | 0,3 |

**TABLE S2.** Taxonomic identity of the 19 core microbiome OTUs across the 142 trees sampled. Taxonomic identification was based on a BLAST against the greengenes database with a minimum cutoff of 50% confidence required for assignment to a given taxonomic group.

**TABLE S3.** Significative associations between bacterial taxonomic groups (a-Phylum, b-Class, c-Order, d-Family and e-OTUs) and tree species (LEfSe analyses). Scores identify which clades have the greatest explanatory power on differences between communities.

a)

| **Bacterial Taxonomic Level** | | **Tree Species** | | **Score** |
| --- | --- | --- | --- | --- |
|  |  | **Angiosperm** | **Gymnosperm** |  |
| Phylum | Bacteroidetes |  | **X** | >3.6 |
|  | Actinobacteria |  | **X** | >3.6 |
|  | Acidobacteria |  | **X** | >3.6 |
|  | OD1 |  | **X** | >3.6 |
|  | Armatimonadetes |  | **X** | >2.4 |
|  | TM7 |  | **X** | >2.4 |
|  | FBP |  | **X** | >2.4 |
|  | Fusobacteria |  | **X** | >2.4 |
|  | TM6 |  | **X** | >1.2 |
|  | Gemmatimonadetes |  | **X** | >1.2 |
|  | Chlamydiae | **X** |  | >1.2 |
|  | Firmicutes | **X** |  | >2.4 |
|  | Proteobacteria | **X** |  | >4.8 |

b)

| **Bacterial Taxonomic Level** | | **Tree Species** | | | | | **Score** |
| --- | --- | --- | --- | --- | --- | --- | --- |
|  |  | **ABBA** | **ACRU** | **ACSA** | **BEPA** | **PIGL** |  |
| **Class** | Actinobacteria |  |  |  |  | **X** | >4.0 |
|  | Gammaproteobacteria |  |  |  |  | **X** | >4.0 |
|  | Saprospirae |  |  |  |  | **X** | >4.0 |
|  | Sphingobacteriia |  |  |  |  | **X** | >3.0 |
|  | Deinococci |  |  |  |  | **X** | >3.0 |
|  | C0119 |  |  |  |  | **X** | >3.0 |
|  | Thermoleophilia |  |  |  |  | **X** | >3.0 |
|  | ML635J_21 |  |  |  |  | **X** | >3.0 |
|  | Fimbriimonadia |  |  |  |  | **X** | >3.0 |
|  | Acidimicrobiia |  |  |  |  | **X** | >2.0 |
|  | Alphaproteobacteria |  |  |  | **X** |  | >5.0 |
|  | Anaerolineae |  |  |  | **X** |  | >3.0 |
|  | Chlamydiia |  |  |  | **X** |  | >3.0 |
|  | Betaproteobacteria |  |  | **X** |  |  | >4.0 |
|  | Cytophagia |  |  | **X** |  |  | >4.0 |
|  | TM7_3 |  | **X** |  |  |  | >3.0 |
|  | Acidobacteriia | **X** |  |  |  |  | >4.0 |
|  | Armanimonadia | **X** |  |  |  |  | >3.0 |
|  | Solibacteres | **X** |  |  |  |  | >3.0 |
|  | SC3 | **X** |  |  |  |  | >3.0 |
|  | Bacilli |  |  | **X** |  |  | >3.0 |

c)

| **Bacterial Taxonomic Level** | | **Tree Species** | | | | | **Score** |
| --- | --- | --- | --- | --- | --- | --- | --- |
|  |  | **ABBA** | **ACRU** | **ACSA** | **BEPA** | **PIGL** |  |
| **Order** | Actinomycetales |  |  |  |  | **X** | >5.0 |
|  | Sphingobacteriales |  |  |  |  | **X** | >4.0 |
|  | Saprospirales |  |  |  |  | **X** | >4.0 |
|  | Deinococcales |  |  |  |  | **X** | >3.0 |
|  | Tremblayales |  |  |  |  | **X** | >3.0 |
|  | Chloroflexales |  |  |  |  | **X** | >3.0 |
|  | Soliubrobacterales |  |  |  |  | **X** | >3.0 |
|  | Fimbriimonadales |  |  |  |  | **X** | >3.0 |
|  | Rhodobacterales |  |  |  |  | **X** | >3.0 |
|  | Rhodospirillales |  |  |  | **X** |  | >5.0 |
|  | Caulobacterales |  |  |  | **X** |  | >3.0 |
|  | Pseudomonales |  |  | **X** |  |  | >5.0 |
|  | Burkholderiales |  |  | **X** |  |  | >4.0 |
|  | Cytophagales |  |  | **X** |  |  | >4.0 |
|  | Acidimicrobiales |  |  | **X** |  |  | >3.0 |
|  | Lactobacillales |  |  | **X** |  |  | >3.0 |
|  | Xanthomonadales |  | **X** |  |  |  | >3.0 |
|  | Alteromonadales |  | **X** |  |  |  | >3.0 |
|  | Sphingomonadales | **X** |  |  |  |  | >5.0 |
|  | Acidobacteriales | **X** |  |  |  |  | >4.0 |
|  | AKIW874 | **X** |  |  |  |  | >3.0 |
|  | Solibacterales | **X** |  |  |  |  | >3.0 |

d)

| **Bacterial Taxonomic Level** | | **Tree Species** | | | | | **Score** |
| --- | --- | --- | --- | --- | --- | --- | --- |
|  |  | **ABBA** | **ACRU** | **ACSA** | **BEPA** | **PIGL** |  |
| **Family** | Chitinophagaceae |  |  |  |  | **X** | >4.0 |
|  | Deinococcaceae |  |  |  |  | **X** | >3.0 |
|  | Sphingobacteriaceae |  |  |  |  | **X** | >3.0 |
|  | Micromonosporaceae |  |  |  |  | **X** | >3.0 |
|  | FFCH7168 |  |  |  |  | **X** | >3.0 |
|  | AK1AB1_02E |  |  |  |  | **X** | >3.0 |
|  | Dermacoccaceae |  |  |  |  | **X** | >3.0 |
|  | Myxococcaceae |  |  |  |  | **X** | >3.0 |
|  | Xanthobacteraceae |  |  |  |  | **X** | >3.0 |
|  | Haliangiaceae |  |  |  |  | **X** | >3.0 |
|  | Tremblayaceae |  |  |  |  | **X** | >3.0 |
|  | Bradyrhizobiaceae |  |  |  |  | **X** | >3.0 |
|  | Nocardioidaceae |  |  |  |  | **X** | >3.0 |
|  | Fimbriimonadaceae |  |  |  |  | **X** | >3.0 |
|  | 0319_6G20 |  |  |  |  | **X** | >3.0 |
|  | Patulibacteraceae |  |  |  |  | **X** | >2.0 |
|  | Alcaligenacea |  |  |  |  | **X** | >2.0 |
|  | Mycobacteriaceae |  |  |  |  | **X** | >2.0 |
|  | Sporichthyaceae |  |  |  |  | **X** | >2.0 |
|  | Acetobacteraceae |  |  |  | **X** |  | >5.0 |
|  | Rhodospirillaceae |  |  |  | **X** |  | >3.0 |
|  | Parachlamydiaceae |  |  |  | **X** |  | >3.0 |
|  | Legionellaceae |  |  |  | **X** |  | >2.0 |
|  | Erythrobacteraceae |  |  |  | **X** |  | >2.0 |
|  | Oxalobacteraceae |  |  | **X** |  |  | >4.0 |
|  | Cytophagaceae |  |  | **X** |  |  | >4.0 |
|  | Microbacteriaceae |  |  | **X** |  |  | >4.0 |
|  | Pseudomonadaceae |  |  | **X** |  |  | >4.0 |
|  | C111 |  |  | **X** |  |  | >3.0 |
|  | Corynebacteriaceae |  |  | **X** |  |  | >3.0 |
|  | Leuconostocaceae |  |  | **X** |  |  | >3.0 |
|  | Bacillaceae |  |  | **X** |  |  | >3.0 |
|  | Kineosporiaceae |  |  | **X** |  |  | >3.0 |
|  | Nocardiaceae |  |  | **X** |  |  | >3.0 |
|  | Beutenbergiaceae |  |  | **X** |  |  | >3.0 |
|  | Intrasporangiaceae |  |  | **X** |  |  | >3.0 |
|  | Halomonadaceae |  |  | **X** |  |  | >3.0 |
|  | Micrococcaceae |  |  | **X** |  |  | >3.0 |
|  | Aurantimonadaceae |  |  | **X** |  |  | >3.0 |
|  | Methylocystaceae |  | **X** |  |  |  | >5.0 |
|  | Beijerinckiaceae |  | **X** |  |  |  | >4.0 |
|  | Aerococcaceae |  | **X** |  |  |  | >4.0 |
|  | Ruminococcaceae |  | **X** |  |  |  | >3.0 |
|  | Geodermatophilaceae |  | **X** |  |  |  | >3.0 |
|  | Conexibacteraceae |  | **X** |  |  |  | >2.0 |
|  | Acidobacteriaceae | **X** |  |  |  |  | >4.0 |
|  | Frankiaceae | **X** |  |  |  |  | >4.0 |
|  | Gordoniaceae | **X** |  |  |  |  | >3.0 |
|  | Ellin122 | **X** |  |  |  |  | >3.0 |
|  | Bartonellaceae | **X** |  |  |  |  | >3.0 |
|  | Pseudocardiaceae | **X** |  |  |  |  | >3.0 |
|  | Streptomycetaceae | **X** |  |  |  |  | >3.0 |
|  | Solibacteraceae | **X** |  |  |  |  | >3.0 |
|  | Phyllobacteriaceae | **X** |  |  |  |  | >3.0 |

| **Bacterial Taxonomic Level** | | **Tree Species** | | | | | **Score** |
| --- | --- | --- | --- | --- | --- | --- | --- |
|  |  | **ABBA** | **ACRU** | **ACSA** | **BEPA** | **PIGL** |  |
| **Species** | *Phytohabitans suffuscus* |  |  |  |  | **X** | >4.0 |
|  | *Mucilaginibacter daejeonensis* |  |  |  |  | **X** | >4.0 |
|  | *Tremblaya phenacola* |  |  |  |  | **X** | >3.0 |
|  | *Rhodospirillum rubrum* |  |  |  |  | **X** | >3.0 |
|  | *Chitinilyticum aquatile* |  |  |  |  | **X** | >2.0 |
|  | *Tanticharoenia sakaeratensis* |  |  |  | **X** |  | <4.0 |
|  | *Neoasaia chiangmaiensis* |  |  |  | **X** |  | <4.0 |
|  | *Gluconacetobacter diazotrophicus* |  |  |  | **X** |  | >3.0 |
|  | *Inquilinus limosus* |  |  |  | **X** |  | >3.0 |
|  | *Liberibacter crescens BT-1* |  |  |  | **X** |  | >2.0 |
|  | *Pseudomonadas fragi* |  |  | **X** |  |  | >5.0 |
|  | *Methylobacterium adhaesivum* |  |  | **X** |  |  | >4.0 |
|  | *Syntrichia ruralis* |  |  | **X** |  |  | >3.0 |
|  | *Methylobacterium organophilum* |  |  | **X** |  |  | >3.0 |
|  | *Sphingomonas echinoides* |  |  | **X** |  |  | >3.0 |
|  | *Pseudomonadas stutzeri* |  |  | **X** |  |  | >3.0 |
|  | *Mycobacterium vaccae* |  |  | **X** |  |  | >3.0 |
|  | *Acinetobacter rhizosphaerae* |  |  | **X** |  |  | >3.0 |
|  | *Pseudoclavibacter helvolus* |  |  | **X** |  |  | >3.0 |
|  | *Salana mutivorans* |  |  | **X** |  |  | >3.0 |
|  | *Janthinobacterium lividum* |  |  | **X** |  |  | >3.0 |
|  | *Methylobacterium mesophilicum* |  |  | **X** |  |  | >2.0 |
|  | *Lysobacter brunescens* |  | **X** |  |  |  | >3.0 |
|  | *Sphingobacterium mizutaii* |  | **X** |  |  |  | >3.0 |
|  | *Sphingomonas changbaiensis* |  | **X** |  |  |  | >2.0 |
|  | *Sphingomonas wittichii* | **X** |  |  |  |  | >5.0 |
|  | *Kaistibacter ginsenosidimutans* | **X** |  |  |  |  | >4.0 |
|  | *Methylovirgula ligni* | **X** |  |  |  |  | >3.0 |
|  | *Sphingomonas yabuuchiae* | **X** |  |  |  |  | >3.0 |
|  | *Novosphingobium nitrogenifigens* | **X** |  |  |  |  | >3.0 |
|  | *Rhodococcus equi* | **X** |  |  |  |  | >3.0 |
|  | *Sphingobacterium faecium* | **X** |  |  |  |  | >3.0 |
|  | *Chitinimonas koreensis* | **X** |  |  |  |  | >2.0 |

**e)**

**f)**

| **Bacterial Taxonomic Level** | | **Tree Species** | | | | | **Score** |
| --- | --- | --- | --- | --- | --- | --- | --- |
|  |  | **ABBA** | **ACRU** | **ACSA** | **BEPA** | **PIGL** |  |
| **OTUs** | 1933 |  |  |  |  | **X** | >2.5 |
|  | 3670 | **X** |  |  |  |  | >2.0 |
|  | 8742 |  |  | **X** |  |  | >2.5 |
|  | 38943 |  | **X** |  |  |  | >2.0 |

**TABLE S4.** Significative associations between bacterial taxonomic groups (a-Phylum, b-Class, c-Order, d-Family and e-OTUs) with tree species classified between angiosperms and gymnosperms (LEfSe analyses). Scores identify which clades have the greatest explanatory power on differences between communities.

| **Bacterial Taxonomic Level** | | **Tree Species** | | **Score** |
| --- | --- | --- | --- | --- |
|  |  | **Angiosperm** | **Gymnosperm** |  |
| Phylum | Bacteroidetes |  | **X** | >3.6 |
|  | Actinobacteria |  | **X** | >3.6 |
|  | Acidobacteria |  | **X** | >3.6 |
|  | OD1 |  | **X** | >3.6 |
|  | Armatimonadetes |  | **X** | >2.4 |
|  | TM7 |  | **X** | >2.4 |
|  | FBP |  | **X** | >2.4 |
|  | Fusobacteria |  | **X** | >2.4 |
|  | TM6 |  | **X** | >1.2 |
|  | Gemmatimonadetes |  | **X** | >1.2 |
|  | Chlamydiae | **X** |  | >1.2 |
|  | Firmicutes | **X** |  | >2.4 |
|  | Proteobacteria | **X** |  | >4.8 |

| **Bacterial Taxonomic Level** | | **Tree Species** | | **Score** |
| --- | --- | --- | --- | --- |
|  |  | **Angiosperm** | **Gymnosperm** |  |
| Class | Actinobacteria |  | **X** | >3.6 |
|  | Acidobacteriia |  | **X** | >3.6 |
|  | Saprospirae |  | **X** | >3.6 |
|  | Deltaproteobacteria |  | **X** | >3.6 |
|  | Sphingobacteriia |  | **X** | >3.6 |
|  | Armatomonadia |  | **X** | >2.4 |
|  | MB_A2_108 |  | **X** | >2.4 |
|  | Solibacteres |  | **X** | >2.4 |
|  | TM7_3 |  | **X** | >2.4 |
|  | Spartobacteria |  | **X** | >2.4 |
|  | Thermoleophilia |  | **X** | >2.4 |
|  | Fimbriimonadia |  | **X** | >2.4 |
|  | Acidimicrobiia |  | **X** | >2.4 |
|  | TM7_1 |  | **X** | >2.4 |
|  | Fusobacteriia |  | **X** | >2.4 |
|  | SC3 |  | **X** | >2.4 |
|  | SJA_4 |  | **X** | >1.2 |
|  | Chlamydiia | **X** |  | >1.2 |
|  | TK10 | **X** |  | >1.2 |
|  | DA052 | **X** |  | >1.2 |
|  | Clostridia | **X** |  | >1.2 |
|  | Pedosphaerae | **X** |  | >2.4 |
|  | Anaerolineae | **X** |  | >2.4 |
|  | Ktedonobacteria | **X** |  | >2.4 |
|  | Bacilli | **X** |  | >2.4 |
|  | Gammaproteobacteria | **X** |  | >3.6 |
|  | Alphaproteobacteria | **X** |  | >3.6 |

| **Bacterial Taxonomic Level** | | **Tree Species** | | **Score** |
| --- | --- | --- | --- | --- |
|  |  | **Angiosperm** | **Gymnosperm** |  |
| Order | Sphingomonadales |  | **X** | >4.8 |
|  | Actinomycetales |  | **X** | >3.6 |
|  | Acidobacteriales |  | **X** | >3.6 |
|  | Sphingobacteriales |  | **X** | >3.6 |
|  | Saprospirales |  | **X** | >3.6 |
|  | Tremblayales |  | **X** | >3.6 |
|  | Chloroflexales |  | **X** | >3.6 |
|  | Neisseriales |  | **X** | >2.4 |
|  | Bdellovibrionales |  | **X** | >2.4 |
|  | Solibacterales |  | **X** | >2.4 |
|  | Chthoniobacterales |  | **X** | >2.4 |
|  | Fimbriimonadales |  | **X** | >2.4 |
|  | Solibrobacterales |  | **X** | >2.4 |
|  | Desulfuromonadales |  | **X** | >2.4 |
|  | 0319_6G20 |  | **X** | >2.4 |
|  | Rhodobacterales |  | **X** | >1.2 |
|  | B07_WMSP1 | **X** |  | >1.2 |
|  | Clostridiales | **X** |  | >1.2 |
|  | Moraxellaceae | **X** |  | >2.4 |
|  | A21b | **X** |  | >2.4 |
|  | Lactobacillales | **X** |  | >2.4 |
|  | Alteromonadales | **X** |  | >2.4 |
|  | Ktedonobacterales | **X** |  | >2.4 |
|  | Oceanospirillales | **X** |  | >2.4 |
|  | Bacillales | **X** |  | >2.4 |
|  | Enterobacteriales | **X** |  | >3.6 |
|  | Rickettsiales | **X** |  | >3.6 |
|  | Rhizobiales | **X** |  | >3.6 |
|  | Pseudomonadales | **X** |  | >4.8 |

| **Bacterial Taxonomic Level** | | **Tree Species** | | **Score** |
| --- | --- | --- | --- | --- |
|  |  | **Angiosperm** | **Gymnosperm** |  |
| Family | Sphingomonadaceae |  | **X** | >3.6 |
|  | Acidobacteriaceae |  | **X** | >3.6 |
|  | Frankiaceae |  | **X** | >3.6 |
|  | Chitinophagaceae |  | **X** | >3.6 |
|  | Sphingobacteriaceae |  | **X** | >3.6 |
|  | Comamonadaceae |  | **X** | >3.6 |
|  | Pelobacteraceae |  | **X** | >3.6 |
|  | FFCH7168 |  | **X** | >3.6 |
|  | Jonesiaceae |  | **X** | >2.4 |
|  | Chrloroflexaceae |  | **X** | >2.4 |
|  | Syntrophaceae |  | **X** | >2.4 |
|  | Heliobacteriaceae |  | **X** | >2.4 |
|  | Bdvellovibrionaceae |  | **X** | >2.4 |
|  | Solibacteracea |  | **X** | >2.4 |
|  | Haliangiaceae |  | **X** | >2.4 |
|  | Tremblayaceae |  | **X** | >2.4 |
|  | Bartonellaceae |  | **X** | >2.4 |
|  | Chthoniobacteraceae |  | **X** | >2.4 |
|  | Gordoniaceae |  | **X** | >2.4 |
|  | Pseudonocardiaceae |  | **X** | >2.4 |
|  | Brazyrhizobiaceae |  | **X** | >2.4 |
|  | Fimbriimonadaceae |  | **X** | >2.4 |
|  | Streptomonadaceae |  | **X** | >2.4 |
|  | Streptomycetaceae |  | **X** | >2.4 |
|  | Nakamurellaceae |  | **X** | >2.4 |
|  | Sinobacteraceae |  | **X** | >2.4 |
|  | AKIW874 |  | **X** | >2.4 |
|  | Hyphomonadaceae |  | **X** | >2.4 |
|  | Hyphomicrobiaceae |  | **X** | >2.4 |
|  | Solirubrobacteraceae |  | **X** | >2.4 |
|  | Patulibacteraceae |  | **X** | >2.4 |
|  | AK1AB1_02E |  | **X** | >2.4 |
|  | Phyllobacteriaceae |  | **X** | >2.4 |
|  | Myxococcaceae |  | **X** | >2.4 |
|  | Rhodobiaceae |  | **X** | >2.4 |
|  | Rhodospirillaceae |  | **X** | >1.2 |
|  | Coxiellaceae |  | **X** | >1.2 |
|  | Xanthobacteraceae |  | **X** | >1.2 |
|  | Mycobacteriaceae |  | **X** | >1.2 |
|  | Conexibacteraceae |  | **X** | >1.2 |
|  | Parachlamydiaceae |  | **X** | >1.2 |
|  | Koribacteraceae | **X** |  | >1.2 |
|  | Gaiellaceae | **X** |  | >1.2 |
|  | Erythrobacteraceae | **X** |  | >1.2 |
|  | Aurantimonadaceae | **X** |  | >1.2 |
|  | FFCH4570 | **X** |  | >1.2 |
|  | Williamsiaceae | **X** |  | >1.2 |
|  | Lactobacillaceae | **X** |  | >1.2 |
|  | Staphylococcaceae | **X** |  | >1.2 |
|  | Shewanellaceae | **X** |  | >2.4 |
|  | Halomonadaceae | **X** |  | >2.4 |
|  | Corynebacteriaceae | **X** |  | >2.4 |
|  | Peptostreptococcaceae | **X** |  | >2.4 |
|  | Clostridiaceae | **X** |  | >2.4 |
|  | EB1003 | **X** |  | >2.4 |
|  | Micrococcaceae | **X** |  | >2.4 |
|  | Bacillaceae | **X** |  | >2.4 |
|  | Streptococcaceae | **X** |  | >2.4 |
|  | Alicyclobacillaceae | **X** |  | >2.4 |
|  | Planococcaceae | **X** |  | >2.4 |
|  | Leuconostocaceae | **X** |  | >2.4 |
|  | Brevibacteriaceae | **X** |  | >2.4 |
|  | Dermatophilaceae | **X** |  | >2.4 |
|  | Aerococcaceae | **X** |  | >2.4 |
|  | Ruminococcaceae | **X** |  | >2.4 |
|  | Piscirikettsiaceae | **X** |  | >2.4 |
|  | AKYG885 | **X** |  | >2.4 |
|  | Dolo_23 | **X** |  | >2.4 |
|  | Prevotellaceae | **X** |  | >2.4 |
|  | Burkholderiaceae | **X** |  | >2.4 |
|  | Pseudomonadaceae | **X** |  | >3.6 |
|  | Enterobacteriaceae | **X** |  | >3.6 |
|  | Oxalobacteraceae | **X** |  | >3.6 |
|  | Methylocystaceae | **X** |  | >3.6 |

| **Bacterial Taxonomic Level** | | **Tree Species** | | **Score** |
| --- | --- | --- | --- | --- |
|  |  | **Angiosperm** | **Gymnosperm** |  |
| Species | wittichi |  | **X** | >4.8 |
|  | suffuscus |  | **X** | >3.6 |
|  | elongata |  | **X** | >3.6 |
|  | ginsenosidimutans |  | **X** | >3.6 |
|  | daejeonensis |  | **X** | >3.6 |
|  | phenacola |  | **X** | >3.6 |
|  | Ellin122 |  | **X** | >3.6 |
|  | ligni |  | **X** | >2.4 |
|  | ochracea |  | **X** | >2.4 |
|  | terrae |  | **X** | >2.4 |
|  | yabuuchiae |  | **X** | >2.4 |
|  | nitrogenifigens |  | **X** | >2.4 |
|  | atsumiense |  | **X** | >2.4 |
|  | acidophilus |  | **X** | >2.4 |
|  | fulvum |  | **X** | >2.4 |
|  | elegans |  | **X** | >2.4 |
|  | equi |  | **X** | >2.4 |
|  | faceium |  | **X** | >2.4 |
|  | bethesdensis |  | **X** | >2.4 |
|  | moabensis |  | **X** | >2.4 |
|  | stellata |  | **X** | >2.4 |
|  | biprosthecium |  | **X** | >2.4 |
|  | gracilis |  | **X** | >2.4 |
|  | leptocrescens |  | **X** | >1.2 |
|  | borealis |  | **X** | >1.2 |
|  | aquatile |  | **X** | >1.2 |
|  | azotifigens |  | **X** | >1.2 |
|  | globiformis |  | **X** | >1.2 |
|  | ochraceum |  | **X** | >1.2 |
|  | plantarum |  | **X** | >1.2 |
|  | rubrum |  | **X** | >1.2 |
|  | koreensis |  | **X** | >1.2 |
|  | piscis |  | **X** | >1.2 |
|  | caeruleus |  | **X** | >1.2 |
|  | rosea |  | **X** | >1.2 |
|  | changbaiensis |  | **X** | >1.2 |
|  | intestinalis | **X** |  | >1.2 |
|  | gibsonii | **X** |  | >1.2 |
|  | grandifolia | **X** |  | >1.2 |
|  | bowmanii | **X** |  | >1.2 |
|  | luteus | **X** |  | >1.2 |
|  | odysseyi | **X** |  | >1.2 |
|  | mendocina | **X** |  | >1.2 |
|  | subarcticum | **X** |  | >1.2 |
|  | versatilis | **X** |  | >1.2 |
|  | mirabilis | **X** |  | >1.2 |
|  | flexus | **X** |  | >1.2 |
|  | ginsengi | **X** |  | >2.4 |
|  | jejuensis | **X** |  | >2.4 |
|  | mixta | **X** |  | >2.4 |
|  | stutzeri | **X** |  | >2.4 |
|  | mitochondria | **X** |  | >2.4 |
|  | helvolus | **X** |  | >2.4 |
|  | Wolbachia endosymb. of C. parallelus | **X** |  | >2.4 |
|  | crescensBT_1 | **X** |  | >2.4 |
|  | aureus | **X** |  | >2.4 |
|  | pycnocarpon | **X** |  | >2.4 |
|  | vaccae | **X** |  | >2.4 |
|  | aerolata | **X** |  | >2.4 |
|  | rifensis | **X** |  | >2.4 |
|  | Ktedonobacteraceae | **X** |  | >2.4 |
|  | algae | **X** |  | >2.4 |
|  | morganii | **X** |  | >2.4 |
|  | guillouiae | **X** |  | >2.4 |
|  | intestini | **X** |  | >2.4 |
|  | diazotrophicus | **X** |  | >2.4 |
|  | ruralis | **X** |  | >3.6 |
|  | adhaesivum | **X** |  | >3.6 |
|  | agglomerans | **X** |  | >3.6 |
|  | cladoniiphilus | **X** |  | >3.6 |
|  | fragi | **X** |  | >4.8 |

**Table S5.** Taxonomic and functional trait information of the five tree species used in this study. Sources for functional trait information are described in the main text.

*Drought tolerance and shade tolerance are two indexes going from one (non-tolerance) to 5 (max-tolerance).

| **Division** | **Family** | **Species** | **Drought**  **tolerance*** | **Hmax**  **(m)** | **Nmass**  **(%)** | **Seed**  **mass**  **(mg)** | **Shade**  **tolerance*** | **SLA**  **(m^2^/g)** | **WD**  **(g/cm^3^)** |
| --- | --- | --- | --- | --- | --- | --- | --- | --- | --- |
| Angiosperm | *Aceraceae* | *Acer rubrum* | 1,8 | 25 | 1,91 | 20 | 3,4 | 0,0141 | 0,49 |
|  |  | *Acer saccharum* | 2,3 | 35 | 1,83 | 65 | 4,8 | 0,0142 | 0,56 |
|  | *Betulaceae* | *Betula papyrifera* | 2 | 25 | 2,31 | 0,33 | 1,5 | 0,0128 | 0,48 |
| Gymnosperm | *Pinaceae* | *Abies balsamea* | 1 | 25 | 1,66 | 7,6 | 5 | 0,0066 | 0,34 |
|  |  | *Picea glauca* | 2,9 | 25 | 1,28 | 2,15 | 4,2 | 0,0033 | 0,35 |


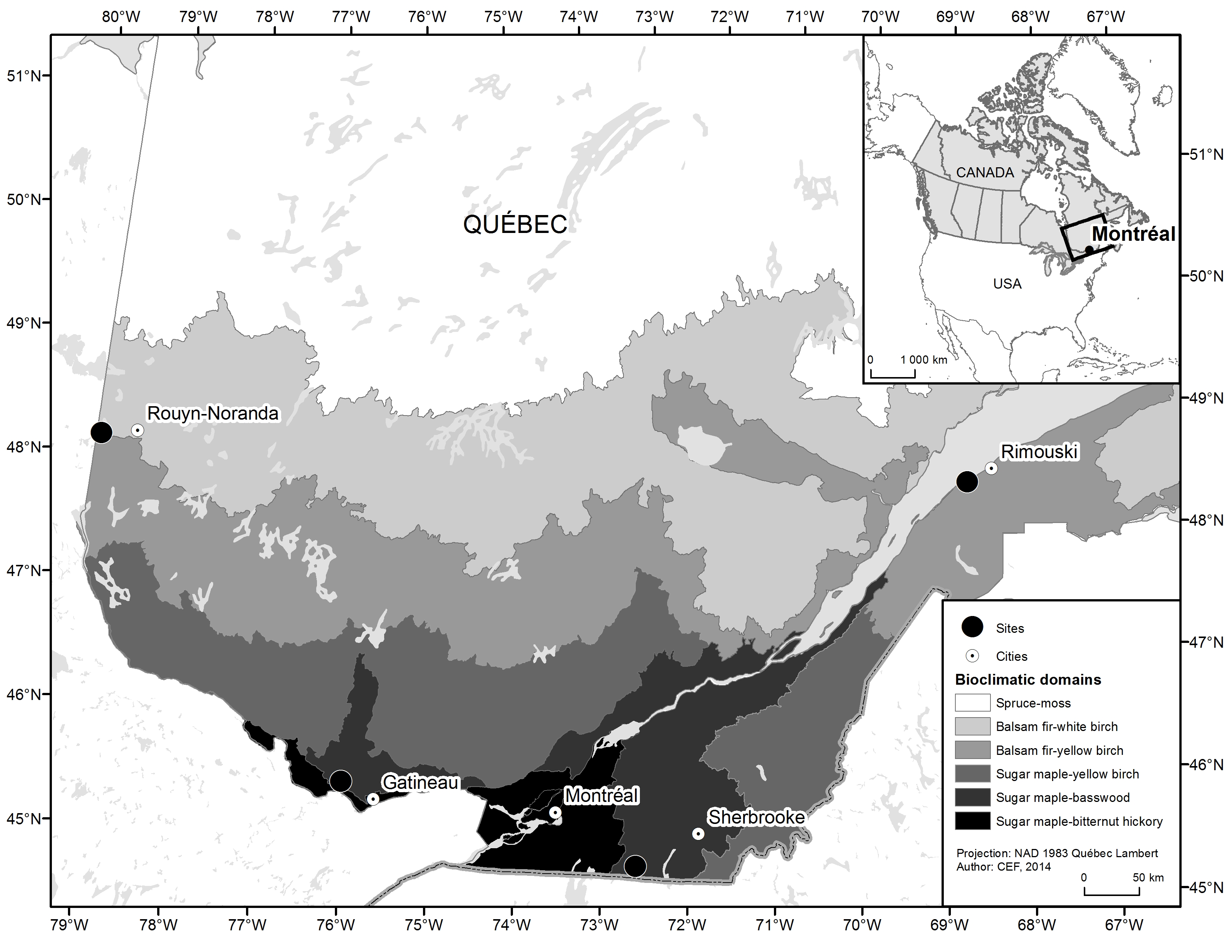


**FIGURE S1.** Location of the four sites sampled during summer 2013 across the temperate forest of Quebec’s province.

**FIGURE S2.** Collector’s curve (mean 95% confidence interval) of bacterial phyllosphere operational taxonomic units (OTUs; 97% sequence similarity cut-off) richness versus number of trees sampled in the temperate forest in 2013.
